# Physical activity and exercise for older people living with HIV – the perceptions of health care professionals

**DOI:** 10.1080/17290376.2025.2590225

**Published:** 2025-11-19

**Authors:** Levin Chetty, Saul Cobbing, Verusia Chetty

**Affiliations:** School of Health Sciences, University of KwaZulu-Natal, Durban, South Africa

**Keywords:** Ageing, HIV, physical activity, exercise

## Abstract

As people age with HIV, the prescription of physical activity should be included as part of their comprehensive medical management. The higher prevalence of comorbidities, polypharmacy, drug interactions and end-stage complications in this specific population requires a multidisciplinary health care approach. This study adopted a phenomenological, qualitative design, using a process of in-depth focus group interviews in order to understand the perceptions of healthcare workers regarding physical activity and exercise for older people living with HIV (OPLWH). Fifteen healthcare professionals who are actively involved in the daily treatment and care of OPLWH at a primary health care facility in KwaZulu-Natal, South Africa voluntarily participated in the study. Three overarching themes emerged from the discussions, namely factors influencing exercise adherence; general considerations for exercise programmes for OPLWH; and recommendations for exercise prescription for OPLWH. This research highlights the importance of the multidisciplinary healthcare team who are at the forefront of care. Continuing assessment of OPLWH, and a suitably conducive environment, need to be taken into consideration when developing and implementing a suitable programme for exercise and physical activity for OPLWH. Challenges such as the stigma associated with HIV, and associated comorbidities related to ageing, need to be addressed when structuring an intervention programme that is sustainable in resource-poor communities.

## Introduction

The dramatic transformation that the world of healthcare has experienced since the advent of the HIV pandemic is continuing to evolve (Bekker et al., 2020). What initially began as a rapidly terminal disease is now a chronic and manageable condition, with noticeably steady increases in life expectancy (Scandlyn, 2000). In the era of antiretroviral therapy (ART), the life expectancy of people living with HIV (PLHIV) is approaching that of the general population (Kaplan-Lewis, Aberg, & Lee, 2017). Predictive modelling suggests that the life expectancy for HIV-infected persons in their 20s, and just starting ART, is only three years less than for their HIV-uninfected counterparts (Wandeler, Johnson, & Egger, 2016).

Medical advances, and the resultant improvements in the lives of PLHIV, have produced a new demographic and subset of older adults ageing with the virus, which demands a prompt response in both clinical practice and research agendas (Guaraldi, Milic, & Mussini, 2019). Much of the published literature under-represents older people living with HIV (OPLWH), and only recently has there been sufficient consensus to develop research that seeks to explore healthcare interventions for this specific population (Rodés, Cadiñanos, Esteban-Cantos, Rodríguez-Centeno, & Arribas, 2022).

OPLWH share many of the same health concerns as the general population aged 50 years and older (Guaraldi et al., 2011). Multiple chronic diseases or conditions; the use of multiple medications; changes in physical and cognitive abilities; and increased vulnerability to stressors, are of major concern (Balderson et al., 2013). Furthermore, OPLWH experience comorbidities at an accelerated rate as compared to their HIV negative counterparts (Gabuzda et al., 2020). In addition, while effective HIV treatment has decreased the likelihood of AIDS-defining illnesses among OPLWH, many HIV-associated non-AIDS conditions occur frequently in OPLWH, such as cardiovascular, metabolic and musculoskeletal complications (Cardoso et al., 2013).

Regular physical activity and exercise are important for PLHIV, especially OPLWH (Chetty, Cobbing, & Chetty, 2021). As individuals age with HIV, they can become susceptible to multiple health conditions resulting from HIV, ageing and the side effects of medication (Cahill & Valadéz, 2013). The physical, cognitive, mental and social health challenges, coupled with uncertainty about future health, collectively contribute to greater prevalence of disability in OPLWH (Johs et al., 2017). Exercise is safe and can improve physical fitness and mental health in OPLWH (Chung & Lou, 2020). Despite this, OPLWH often have sedentary lifestyles and are more physically inactive than other similar populations with chronic diseases (Safeek et al., 2018). As people age with HIV, the prescription of physical activity should be included as part of their comprehensive medical management (Hand, Lyerly, Jaggers, & Dudgeon, 2009; Mutimura, Stewart, Crowther, Yarasheski, & Cade, 2008). Healthcare and prevention guidelines tailored to OPLWH are needed. The higher prevalence of comorbidities, polypharmacy, drug interactions and end-stage complications, such as neuropsychiatric, musculoskeletal, cardiovascular, pulmonary and endocrine function in this specific population requires a multidisciplinary approach (Gabuzda et al., 2020). This study draws on a set of community-based focus-group interviews (FGIs) with healthcare professionals in order to understand their perceptions of physical activity and exercise for OPLWH. Exploring their attitudes, and understanding their perceptions about physical activity and exercise for this specific population, will also assist in distinguishing between the viewpoints of various healthcare professionals that offer holistic care to OPLWH.

## Methods

### Design

The study adopted a phenomenological, qualitative design, using a process of in-depth focus group interviews in order to understand the perceptions of healthcare workers regarding physical activity and exercise for OPLWH. The authors believe that gaining insight into the experiences and perceptions of healthcare professionals, especially those at the forefront of providing care to OPLWH, is a critical step in the development and implementation of physical activity approaches and exercise prescriptions for OPLWH. Similarly, literature reports that successful and sustainable implementation of novel approaches to HIV care as one gets older requires support at both the local and national levels of health care, and must include healthcare providers at both levels (Doornebosch, Smaling, & Achterberg, 2022; Erlandson & Karris, 2019; Hall & Weaver, 2001). Hence, we utilised this phenomenological approach, using an informal interactive interview process (Cypress, 2018) in order to develop a deeper understanding of healthcare workers’ experience of physical activity and exercise for OPLWH.

### Participant selection

Participants for the study were purposively sampled using a maximum variation sampling technique. This method allowed the inclusion of the multidisciplinary healthcare team of professionals (both male and female) who were involved in the treatment and care of OPLWH at the study setting. In accordance with the inclusion criteria of the study, health professionals were selected if they were employed at the study setting for a period of five years or more, and were involved in the day to day care of OPLWH. Following a rigorous process of emailing and telephonic invitation to participants, fifteen (15) out of twenty-three (23) healthcare professionals voluntarily consented to participate in our study.

### Study setting and research team

The focus group discussions with healthcare professionals were conducted in a private seminar room in the physiotherapy department within the healthcare facility. Our healthcare professionals participated in three focus group discussion sessions that lasted between sixty to ninety minutes each, and each group included seven to eight participants. The study setting, a 200-bed, Level 1 District Hospital, is situated in the outer district of the eThekwini Municipality in the KwaZulu-Natal province of South Africa. It serves a population of approximately one million people and acts as a referral hospital for 16 provincial and municipal primary healthcare clinics, as well as two community health centres (CHCs). Estimates suggest that more than 250 000 people (33%) living in this catchment area are HIV-positive. Our research team was made up of both male and female scientists (with Masters and PhD qualifications), specialising in HIV research and rehabilitation in South Africa and have a long-standing partnership with the healthcare setting. The focus group facilitator had approximately fifteen years of qualitative research expertise in conducting focus group interviews.

### Data collection

The study was conducted from June to November 2022. Informed consent for participation in the study was obtained and permission to use audio recordings was granted by all the healthcare professionals who participated in the study. Each participant was informed that their participation was voluntary, and of their right to withdraw at any stage of the study. No financial remuneration was provided. The discussions were conducted in English. An interviewer and an expert moderator in qualitative research were present during the focus group discussions. The questions remained open and flexible but focused on healthcare workers experience of caring for OPLWH, their understanding of physical activity and exercise for OPLWH, influencers of physical activity and exercise rehabilitation in OPLWH including facilities infrastructure, their view on rehabilitation programmes offered at the facility and perceptions of improving evaluation and monitoring. Finally, questions aimed at healthcare workers recommendations of how they perceived rehabilitation including physical activity and exercise can be improved to offer best care.

### Data analysis

Data was analysed using NVivo thematic analysis software. Responses from the participants were transcribed verbatim, immediately after the focus group discussions. Transcripts were read and re-read by two of the researchers (LC & VC), independently, to gain an understanding of the composite narrative. The third author (SC) mediated discrepancies and all researchers (LC, VC and SC) conceded on the final themes. An inductive approach was used to analyse the data, thereby allowing the researchers to analyse the data using a bottom-up approach, ensuring that codes and themes were derived from the content of the data itself. Braun and Clarke’s open coding process was followed to identify common concepts in the participants’ responses. As outlined by Braun and Clarke, the six-step approach to thematic analysis included: (1) familiarising oneself with the data; (2) the generation of initial codes; (3) searching for themes; (4) reviewing the potential themes; (5) defining and naming the themes; and (6) producing the report (Clarke & Braun, 2017). Member checking was conducted to verify the accuracy of the data transcription. In order to gain a clear, comprehensive view of the phenomenon, the process of method triangulation was used throughout each phase to ensure the credibility and trustworthiness of the data (Cope, 2014). Rigour was also ensured by an extensive audit trail and field notes, and the process was checked by an expert in qualitative research methods (Cresswell & Cresswell, 2018).

## Results

Fifteen healthcare professionals (four doctors; four nurses; four physiotherapists; two occupational therapists; and one psychologist) participated in the face-to-face focus group discussions. All the healthcare professionals who participated in our study were actively involved in the day-to-day care of OPLWH at the facility. The participants demographics are represented in Table 1. Eleven female and four male healthcare professionals ranging in experience from five to seventeen years of HIV care participated in the study.

**Table 1.** Participant demographics.

Health professional	Age	Sex	Ethnicity	Experience (years)
Doctor 1	56	Male	Black	17
Doctor 2	49	Male	Black	10
Doctor 3	52	Male	Black	15
Doctor 4	50	Female	Indian	5
Nurse 1	46	Female	Black	10
Nurse 2	44	Female	Black	9
Nurse 3	53	Female	Black	12
Nurse 4	49	Female	Black	15
Physiotherapist 1	32	Female	Black	12
Physiotherapist 2	26	Female	Indian	5
Physiotherapist 3	29	Female	Indian	8
Physiotherapist 4	27	Male	Indian	6
Occupational Therapist 1	27	Female	White	5
Occupational Therapist 2	28	Female	White	6
Psychologist 1	39	Female	White	10

Three overarching themes emerged from the discussions, namely factors influencing exercise adherence; general considerations for exercise programmes for OPLWH; and recommendations for exercise prescription for OPLWH. The themes, subthemes and illustrative quotes of participants are presented in Table 2.

**Table 2.** Themes, subthemes and illustrative quotes of participants.

THEME 1: FACTORS INFLUENCING EXERCISE ADHERENCE	
Patient comorbidities and exercise	‘Given the fact that they now have an added comorbidity or a sickness that now predisposes them to much more infections than being sick, it is just good for them to be active and exercise. I think structured form of exercise would be of great benefit’. (Physiotherapist, 8 years’ experience) ‘They usually present very late and they have comorbidities, so they will have your arthritis, they will have your hypertension, they will have diabetes and they will have DVTs, which is what we see very commonly with our patients. What’s nice about them is that once you’ve started them on the ARVs, they tend to do very well. And together with exercise, this can only be good’. (Doctor, 10 years’ experience)
Socio-economic status	‘I think accessibility is a big thing. A lot of the patients that come in with HIV, either they’re poor or they’re not working, or ja (yes), I don’t think there’s necessarily any financial stability. So, their economic status, I would say, it does impact on how well they are going to exercise or if they are ever going to turn up for sessions’. (Occupational Therapist, 6 years’ experience) ‘Poor social economic status will affect adherence as well. You can’t say to somebody … oh yeah, you must go to the gym and exercise. They don’t even have a R10.00 to take a taxi. All too often we may only focus on the poor social aspect. We must not forget to also focus on the environment in which they can exercise and policy as well’. (Doctor, 17 years’ experience)
Stigma impeding exercise	‘I think there is definitely a stigma attached to participation in exercise because of confidentiality reasons and also the fear of being judged … but at the same time, being in an environment with people of like similar to you, I feel like maybe it would lessen the feeling of being judged because you are around people who are just like you. I think that they would be more hyper-aware of like being seen by other people or being judged’. (Psychologist, 10 years’ experience) ‘If we do not encourage them, if we do not emphasize that it is important, sometimes they may neglect it. If they have got negative perceptions of their disease, or how the family views them, that might not motivate them to exercise. If they understand their disease and they are willing to modify their lifestyle and to adapt to their treatments, generally they do well’. (Nurse, 12 years’ experience)
THEME 2: GENERAL COMPONENTS FOR EXERCISE PROGRAMMES FOR OPLWH	
Multi-disciplinary approach	‘All health professionals who deal with this population can help each other. Communication is the key … . communicate with each other and know that it’s okay, we can still do so much more to get them exercising.’. (Doctor, 17 years’ experience) ‘If I am honest, there has been a neglect of holistic interventions for older people with HIV. We are responsible for that. I think a lot of involvement from the healthcare professionals is necessary. A lot of members from the MDT should be involved, so that there is attention, kind of spread out everywhere. The patient is then aware that their doctor or nurse or allied health professional agrees that exercise is important for them’. (Doctor, 5 years’ experience)
Suitable exercise environment	‘When there is a facility that one can attend for exercise, there is almost a sense of group belongingness. I am going to a place, I am getting a service, a facility that you are kind of forced to take accountability’. (Nurse, 9 years’ experience) ‘It would depend on how many patients we can fit at a time, like in the rooms as well, because there is limited space here. I think a nice area for the activities to take place would be beneficial, or maybe outside in a large, safe environment’. (Doctor, 17 years’ experience)
Continuing assessment of OPLWH	‘You look at a person, based on their function and things like that, and then I also think, if with HIV obviously, they can be very, very healthy with HIV; but if they’re coming from not such a good baseline, or a lower baseline, actually take that into account and adjust it, based on how they feel confident progressing so that they can continue doing things. We have to provide continuous exercise assessments, using various parameters’. (Nurse, 15 years’ experience) ‘Because they are older you have to consider their vitals, things that tire them, like exercise – They can’t do this, they can’t do that. Then they will need specific exercises, and it would then be wise to split people into their level of function, and continuous monitoring vitals and so forth’. (Doctor, 5 years’ experience)
THEME 3: RECOMMENDATIONS FOR EXERCISE PRESCRIPTION FOR OPLWH	
Type of exercise recommended	‘So, as with any exercise programme, you probably have to have strength, endurance, proprioception, agility – all of those are even more important for HIV’. (Nurse, 9 years’ experience) ‘Aerobic exercises, those that increase the heartrate is very important. Exercises that will promote general wellbeing basically, proper movement of the joints, stretches, general muscle strengthening which can help you to be strong and definitely add to their endurance’. (Physiotherapist, 10 years’ experience) ‘All too often we, as doctors, may only prescribe aerobic activity, like walking. But I think all are important – strength, flexibility, agility and aerobic, especially for this population. They need it, they often present with low strength and endurance levels’. (Doctor, 17 years’ experience)
Frequency and duration of exercise	‘At least twice a week. I know there is a general consensus of three times a week, but given our population dynamics, and the affordability of people to actually come to the centre, I would say two times a week, but ideally three times for approximately 30–45 min’. (Doctor, 15 years’ experience) ‘Obviously we do not expect this population to exercise like their younger counterparts. I would suggest two times a week and then progress to three times a week as they build exercise tolerance, and we should be able to test for improvements with regular assessments. But nothing more than 30 min per session. I read that in an article or somewhere’. (Physiotherapist, 8 years’ experience)
Intensity of exercise	‘I would like to see them begin with low intensity activities, like perhaps 20% of their HR and gradually increase as they progress. We definitely cannot start them at a high intensity level’. (Doctor, 5 years’ experience) ‘A gradual progression in exercise intensity. I’m not too sure how – you guys are the experts in exercise but I think we all agree that it has to be gradually incremental. We cannot have them stay at the same level for too long. I don’t think that will benefit them’. (Doctor, 15 years’ experience)
Group versus individual exercise	‘I think at a group level, exercise would be more beneficial. At a group level, patients may feel like there is a kind of a belongingness. It brings those elements of excitement and fun. Because if they are together, they will see that oh, okay, this age group of mine, okay they can do this, okay I can do it, you see’. (Occupational Therapist, 5 years’ experience) ‘A structured group exercise routine, I think, is long overdue for this older population. It motivates people to carry on doing the exercise for longer, as opposed to an individual doing their own thing. And if you say it is an exercise group for older people and you bring to the fore that they have HIV, it would be more inclusive, it would not be resisted’. (Doctor, 10 years’ experience)
Supervised exercise sessions	‘If we truly want to see long term adherence from patients to these sorts of programmes, then continuous supervision through each session is a must. It will also help if the exercises are structured so none of the patients are doing their own thing. Older people get distracted easily and like to talk, so we have to keep them on track so they don’t lose interest. (Nurse, 9 years’ experience) ‘They can't do this on their own, they need supervised exercise guidance. They need professionals to show them what to do. Maybe when they are familiar with how to do the exercises and how much to do, then we can give them exercises they can do at home’. (Physiotherapist, 12 years’ experience)

Under Theme One, our participants identified patient comorbidities and exercise, socio-economic status, and stigma, as exercise-impeding factors that influence exercise adherence in OPLWH. The concern for many of the healthcare professionals was that comorbidities associated with HIV, together with the very low socio-economic status of patients, as well as the attitudes and mindset of the patients, would not provide sufficient motivation to continue a structured exercise regime for prolonged periods of time.

In Theme Two, the health professionals commented about the general components for exercise programmes for OPLWH. There was consensus that a multidisciplinary approach, a suitable exercise environment, and the continuing assessment of participants, were serious issues that required further exploration to ensure the successful implementation of such a programme for OPLWH. A multidisciplinary team approach was supported by all our health professionals. They agreed that communication between all members of the healthcare team was the best way to ensure buy-in from OPLWH, while some even proposed that they would be happy to participate physically in such programmes with OPLWH.

In Theme Three, our cohort debated the elements of exercise prescription for OPLWH. They identified five sub-themes, namely type of exercise; frequency and duration of exercise; intensity of exercise; group versus individual exercise; and supervised exercise sessions, as critical elements in exercise prescription for OPLWH. All agreed that the prescription must be multimodal and should include components of endurance, strength, balance, and flexibility. Frequency and duration ranged from exercising two-to-three times per week, for anywhere between thirty minutes and an hour. All our health professionals also advocated that prescribing supervised, structured group exercise would be most beneficial for this population.

## Discussion

This is the first study, to our knowledge, that seeks to explore the perceptions of healthcare professionals regarding physical activity and exercise for OPLWH. Findings from our study are partly aligned with previous research conducted on the perceptions of OPLWH about physical activity and exercise (Chetty, Cobbing, & Chetty, 2022). These findings will be discussed under the three themes.

Theme One focused on factors that influence exercise adherence among OPLWH, from the perspective of healthcare workers. Healthcare workers identified patient co-comorbidities and exercise, socio-economic status, and stigma, as exercise-impeding factors that influence exercise adherence in OPLWH. The current challenge in HIV care is focused on successful prevention and treatment of comorbidities due to the negative impact of comorbidities on quality of life (Erlandson & Karris, 2019). Given that the risk of comorbidities is not homogenous, even within various HIV populations, in terms of prevention and management, susceptible HIV groups such as OPLWH, would benefit more from targeted interventions, such as physical activity and exercise (Martínez-Sanz, Serrano-Villar, Vivancos, Rubio, & Moreno, 2022). Natural ageing should also be considered as an influencing factor. In addition, socio-economic status and stigma are of concern and were identified in our paper as inhibiting exercise and physical activity. Stigma is regarded as a significant predictor of physical activity adherence among PLHIV, both in our, and other, studies (Johs, Kellar-guenther, Jankowski, Neff, & Erlandson, 2019; Jones et al., 2012; Rodriguez-moreno, Calderón-cardona, Alberto, Yepes-delgado, & Calderón-cardona, 2024). The double influence of stigma as a result of the virus, coupled with socio-economic status, has been reported in previous studies as having an adverse effect on physical activity adherence in PLHIV in a South African community (Ley, Barrio, & Leach, 2015). Despite the positive effects of physical activity and exercise on physical and mental health, Vancampfort and colleagues point out that participation in physical activity by PLHIV is determined by a number of complex factors, ranging from advanced age, lower educational levels, lower concentration of CD4 cell count, exposure to antiretroviral drugs, presence of lipodystrophy, presence of pain, depression, and opportunistic infections (Vancampfort et al., 2018). The authors further highlighted the need for special attention in older HIV groups to improve adherence to physical activity, especially those with lower educational levels, lipodystrophy and ART. Our cohort of healthcare workers similarly advocated for comprehensive research within a socio-ecological model of care. This included further research into social, environmental and policy guidelines, in order to improve physical activity and exercise adherence levels in OPLWH.

Under Theme Two, our participants believed that a multi-disciplinary approach, suitable exercise environment, and continuing assessment of participants, are vital components for exercise prescription and adherence for OPLWH. The multi-disciplinary team approach in HIV care evolved out of necessity, due to the diverse characteristics and needs of PLHIV, and it is widely accepted as the international standard of care in HIV (Magnus & Castel, 2016; Sherer et al., 2002). The ever increasing burden of caring for patients in palliative care makes it impractical for healthcare professionals to work in silos (Hall & Weaver, 2001). There is sufficient evidence that a multi-disciplinary team approach works in the management of chronic illnesses, such as mental health, diabetes, cancer and heart disease (Chetty & Hanass-Hancock, 2016). However, this approach may be lacking due to medical dominance, poor interprofessional knowledge, poor communication and limited sharing of practical knowledge between healthcare professionals, especially in low to middle income countries (V. Chetty & Maharaj, 2013; Doornebosch et al., 2022). Benagiano and Brosens argues that it gives the patient access to the right team of healthcare professionals who work together to plan the most suitable care option, minimising delays in treatment and referral to services, whilst also transferring consistent and appropriate information to patient and family (Benagiano & Brosens, 2014). The healthcare workers in our paper believed it would be a suitable approach for exercise prescription and adherence in OPLWH. A suitable exercise environment and the continuing assessment of health parameters were recommended in previous studies into OPLWH, which identified similar factors as enablers of long-term participation in physical activity programmes (Chetty et al., 2022). Regular health monitoring of individuals with HIV is encouraged throughout the exercise programme, as is generally recommended for persons with chronic diseases (Thompson, Arena, Riebe, & Pescatello, 2013). Also, maintaining adequate hydration and exercising in a venue with comfortable temperatures and excellent ventilation are just as important, because of potential problems with sweating and temperature regulation that can occur with autonomic dysfunction (Pescatello, Arena, Riebe, & Thompson, 2013).

Under Theme Three, our participants were questioned about their understanding of exercise prescription for OPLWH. The lack of advice about physical activity and exercise parameters from healthcare providers has been widely reported by PLHIV (Chetty et al., 2022; Quigley et al., 2019). The healthcare workers in our paper believed that multimodal exercise prescription was best suited for this population. Multimodal exercise training has been indicated for PLHIV, given its potential to improve several aspects of physical fitness and cardiometabolic risk (Gama et al., 2022; Paz, Farinatti, Lopes, & Borges, 2021; Rodrigues et al., 2018). Recommended frequency and duration ranged from exercising two-to-three times per week, for between thirty minutes and an hour, with gradual, incremental increases in exercise intensity most beneficial. The principles for exercise prescription used for healthy adults can generally apply to PLHIV, with some modifications according to their health status (Bushman, 2022; Garber et al., 2011). Although most studies have demonstrated the beneficial effects of exercise in PLHIV, optimal exercise prescriptions have yet to be identified. Most research studies use exercise intensities ranging from 50% to 75% of heart rate reserve (HRR) without any noticeable complications, while exercise duration has typically ranged from 30 to 60 min per session, with a frequency of at least three days per week (Lindegaard et al., 2008; Lox, McAuley, & Tucker, 1996; Mutimura et al., 2008; Smith et al., 2001; Terry et al., 2006). Finally, the healthcare workers in this study recommended supervised group exercise sessions. Similar findings were found in a paper about an older HIV population who were supportive of supervised group exercise sessions to promote social interaction, increase motivation and encourage greater adherence levels (Chetty et al., 2022; Li et al., 2017; Quigley et al., 2019).

## Conclusion

Physical activity and exercise strategies tailored to OPLWH are essential in the current era of holistic HIV care. This paper highlights the importance of the multidisciplinary healthcare team who are at the forefront of care. Continuing assessment of OPLWH, and a suitably conducive environment, need to be taken into consideration when developing and implementing a suitable programme for exercise and physical activity for OPLWH. Challenges such as the stigma associated with HIV, and associated comorbidities related to ageing, need to be addressed when structuring an intervention programme that is sustainable in resource-poor communities. A limitation of the study is the omission of psychosocial models which were not addressed in the framework. The study specifically focused on physical activity and exercise prescriptions for OPLWH.

## Supplementary Material

Supplemental Material
