# Supplementary material for: Physical activity and exercise for older people living with HIV – the perceptions of health care professionals
Source: SAHARA J. 2025 Nov 19;22(1):2590225. doi: 10.1080/17290376.2025.2590225 (PMC12632215; doi:10.1080/17290376.2025.2590225)
Supplement: Supplemental Material [file RSAH_A_2590225_SM6663.pdf]

UNIVERSITY OF KWAZULU-NATAL™
INYUVESI YAKWAZULU-NATALI

16 September 2021

Mr Levin Chetty (9904071)
School of Health Sciences
Westville

Dear Mr Chetty,

Protocol reference number: BREC/00001358/2020
Project title: Exercise Prescription Guidelines for Older People Living with HIV in South Africa (Phase 2 - Perceptions of Older People Living with HIV and Healthcare Professionals)
Degree Purposes: PhD

RECERTIFICATION APPLICATION APPROVAL NOTICE

**Approved:**	**01 September 2021**
**Expiration of Ethical Approval:**	**30 November 2022**

I wish to advise you that your application for recertification received on 03 September 2021 for the above study has been **noted and approved** by a subcommittee of the Biomedical Research Ethics Committee (BREC).The start and end dates of this period are indicated above.

If any modifications or adverse events occur in the project before your next scheduled review, you must submit them to BREC for review. Except in emergency situations, no change to the protocol may be implemented until you have received written BREC approval for the change.

The committee will be notified of the above at its next meeting to be held on 12 October 2021.

Yours sincerely

……………………………………………………….

Ms A Marimuthu
(for) Prof D Wassenaar
Chair: Biomedical Research Ethics Committee
